# Supplementary material for: Polar meron-antimeron networks in strained and twisted bilayers
Source: Nat Commun. 2023 Mar 24;14:1629. doi: 10.1038/s41467-023-37337-8 (PMC10036565; doi:10.1038/s41467-023-37337-8)
Supplement: Supplementary file 1 — Supplementary Information [file 41467_2023_37337_MOESM1_ESM.pdf]

SUPPLEMENTARY INFORMATION
Polar meron-antimeron networks in strained and twisted bilayers

Daniel Bennett,[1,2,3,*] Gaurav Chaudhary,[2] Robert-Jan Slager,[2] Eric Bousquet,[1] and Philippe Ghosez[1]

[1]*Physique Théorique des Matériaux, QMAT, CESAM, University of Liège, B-4000 Sart-Tilman, Belgium*
[2]*Theory of Condensed Matter Group, Cavendish Laboratory, University of Cambridge, J. J. Thomson Avenue, Cambridge CB3 0HE, United Kingdom*
[3]*John A. Paulson School of Engineering and Applied Sciences, Harvard University, Cambridge, Massachusetts 02138, USA*

(Dated: March 9, 2023)

I. MEASURING POLARIZATION IN CONFIGURATION SPACE

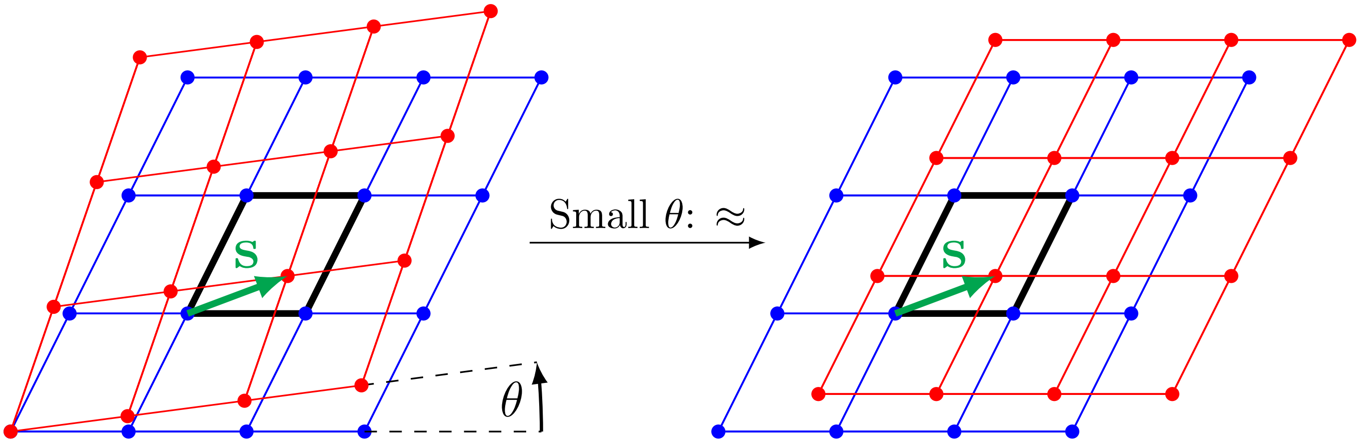

Supplementary FIG. 1. Sketch of how the configuration space mapping is used to estimate the local polarization in a twisted bilayer for small twist angles.

In this section we briefly describe how the configuration space mapping is used to estimate local properties in bilayers with small strains or twist angles. For a more detailed description, see Refs. 1 and 2. We illustrate the problem for the case of twist, considering two hexagonal monolayers (red and blue), each with a single atom in the unit cell, twisted at an angle θ with respect to one another, see Fig. 1. Suppose we want to estimate the local properties in the middle unit cell, marked in black, using first-principles calculations. Using the configuration space mapping, the relative position $\mathbf{s}$ of the red atom in this cell is $\mathbf{s}(\mathbf{r}) = (I - R_\theta^{-1})\mathbf{r}$, modulo any lattice vectors, where $\mathbf{r}$ is the real space position. For small twist angles, we make the approximation that the local changes in environment around the black unit cell are small, and the local properties can be described by a commensurate bilayer with a relative translation $\mathbf{s}$:

$$\mathbf{s} \approx \theta \begin{bmatrix} 0 & -1 \\ 1 & 0 \end{bmatrix} \mathbf{r}\,, \tag{1}$$

see Fig. 1. For a small homogeneous strain η, the equivalent mapping is

$$\mathbf{s} = \eta \mathbf{r}\,. \tag{2}$$

This allows the local properties in strained or twisted bilayers to be parameterized with first-principles calculations using a single commensurate cell of a bilayer, and sliding one layer over the other.

The total polarization, both out-of-plane and in-plane, can then be calculated from Berry phases:

$$\mathbf{P}(\mathbf{s}) = -\frac{1}{2\pi}\frac{e}{V(\mathbf{s})}\sum_{n}^{\text{occ}} \phi_{n,\alpha}(\mathbf{s})\mathbf{a}_\alpha\,, \tag{3}$$

* dbennett@seas.harvard.edu

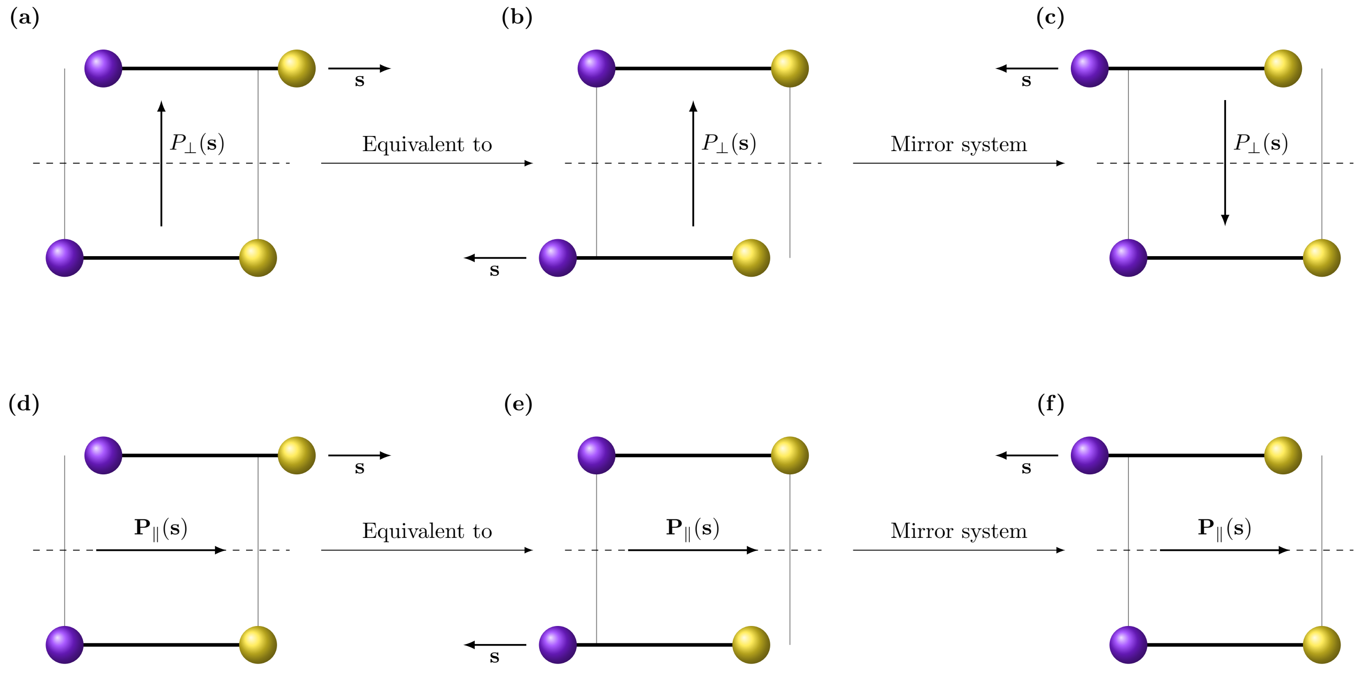

Supplementary FIG. 2. Sketch illustrating the effect of the mirror operation on **(a)-(c)**: the out-of-plane and **(d)-(f)**: the in-plane components of the polarization.

where $V(\mathbf{s}) = Ad(\mathbf{s})$ is the volume of the bilayer, $\mathbf{a}_\alpha$ are the in-plane lattice vectors, and $\phi_{n,\alpha}$ is the Berry phase. The sum is over all occupied bands n. The Berry phase is routinely calculated with most widely-available first-principles density functional theory (DFT) codes:

$$\phi_{n,\alpha}(\mathbf{s}) = \frac{(2\pi)^3}{V(\mathbf{s})} \int_{\mathrm{BZ}} \langle u_{n,\mathbf{k}}(\mathbf{s}) | -i\mathbf{G}_\alpha \cdot \nabla_{\mathbf{k}} | u_{n,\mathbf{k}}(\mathbf{s}) \rangle \, \mathrm{d}\mathbf{k} \, , \tag{4}$$

where $\mathbf{G}_\alpha$ are the reciprocal lattice vectors and $u_{n,\mathbf{k}}(\mathbf{s})$ are the cell-periodic Bloch functions. Eq. (4) is essentially the position of the Wannier center, but normalized by the length of the in-plane lattice vectors and 2π.

The local polarization in the main text was calculated by sliding one layer of hBN over the other, and at each point calculating the total polarization from Berry phases.

Of course, this method of calculating the local polarization in a twisted bilayer is not valid at all twist angles; at larger twist angles, the local changes in the environment cannot be assumed to be constant. We propose two more well-defined methods of measuring the local polarization:

1. By calculating the Wannier centers. The local polarization in each unit cell is then obtained by summing over the Wannier centers in that cell.

2. By integrating the dynamical charges.

In Section IV, we show that method 2 yields the exact same polarization obtained from Berry phases in configuration space. In principle, both of these methods should make it possible to directly calculate the local polarization in a twisted bilayer, although the calculations may be prohibitvely expensive.

II. SYMMETRY OF THE POLARIZATION FIELD IN BILAYER hBN

Using the in-plane mirror symmetry of the AA stacking configuration, we can deduce the forms of the out-of-plane and in-plane polarization in 3R-stacked bilayer hBN.

For bilayer hBN with an AA stacking configuration, there is a mirror symmetry about the plane which is half-way between the two layers, and the out-of-plane polarization is zero. Suppose we translate the top layer by $\mathbf{s}$, generating an out-of-plane

polarization $P_\perp(\mathbf{s})$. This is equivalent to translating the bottom layer by $-\mathbf{s}$. If we now mirror the system about the plane between the two layers, the out-of-plane polarization is inverted. The same configuration can be achieved by sliding the top layer by $-\mathbf{s}$. Therefore we can deduce that

$$P_\perp(\mathbf{s}) = -P_\perp(-\mathbf{s}) \; , \tag{5}$$

i.e. the out-of-plane polarization is odd with respect to in-plane translations. This is illustrated in Figs. 2 (a)-(c).

Repeating this exercise with the in-plane polarization $\mathbf{P}_\parallel(\mathbf{s})$, we find that the in-plane polarization remains the same after applying the mirror symmetry, since it parallel to the mirror plane. Therefore we must have

$$\mathbf{P}_\parallel(\mathbf{s}) = \mathbf{P}_\parallel(-\mathbf{s}) \; , \tag{6}$$

i.e. the in-plane polarization is even with respect to in-plane translations. This is illustrated in Figs. 2 (d)-(f).

III. POLAR MODE PHONON FREQUENCIES IN BILAYER hBN

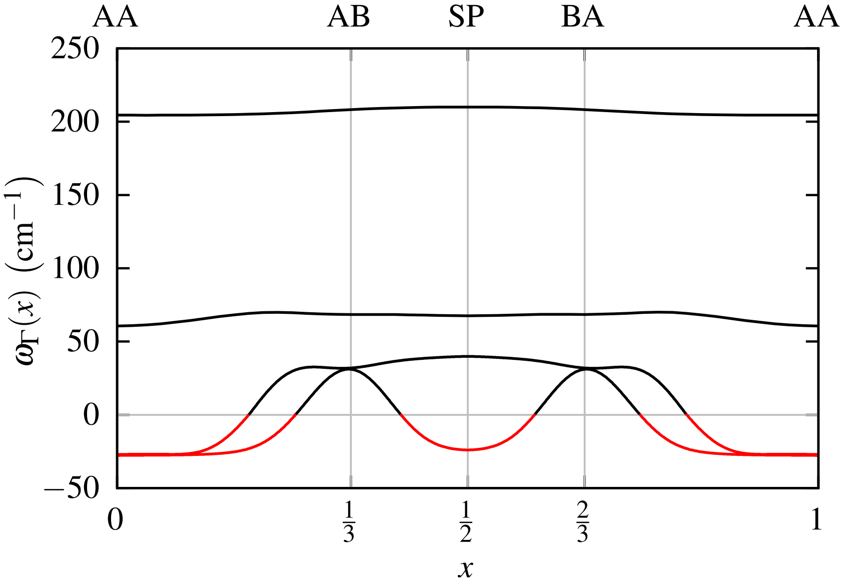

Supplementary FIG. 3. Lowest Gamma phonon frequencies in 3R-stacked bilayer hBN as a function of slide along the configuration space diagonal. The bands are shown in black when the phonons are stable, and red when they are unstable.

IV. DYNAMICAL CHARGES

In order to better understand the physical origin and nature of the in-plane and out-of-plane polarization, we calculated the dynamical charges[3] in bilayer hBN as a function of relative stacking:

$$Z^*_{\kappa,\alpha\beta} = V \frac{\partial P_\alpha}{\partial s_{\kappa,\beta}} \; , \tag{7}$$

i.e. the dipole generated in direction α in response to the unitary displacement of atom κ in direction β. Since the bilayer is a semi-periodic system, Eq. (7) is measured at zero electric field for the in-plane responses, and with open circuit boundary conditions for the out-of-plane responses. Typically, Eq. (7) is symmetric or has a negligible anti-symmetric part, and can be diagonalized. The effective charge of each atom can therefore be thought of as an ellipsoid, with lengths and orientations of the axes determined by the eigenvalues and eigenvectors[3]. The change in polarization from a reference configuration at $\mathbf{s} = 0$ (AA stacking) to a general configuration at $\mathbf{s}$ can be measured by integrating the dynamical charges:

$$P_\alpha(\mathbf{s}) = \frac{1}{V(\mathbf{s})} \int_0^{\mathbf{s}} Z^*_{\kappa,\alpha\beta}(\mathbf{s}')\, \mathrm{d}s'_{\kappa,\beta} \; . \tag{8}$$

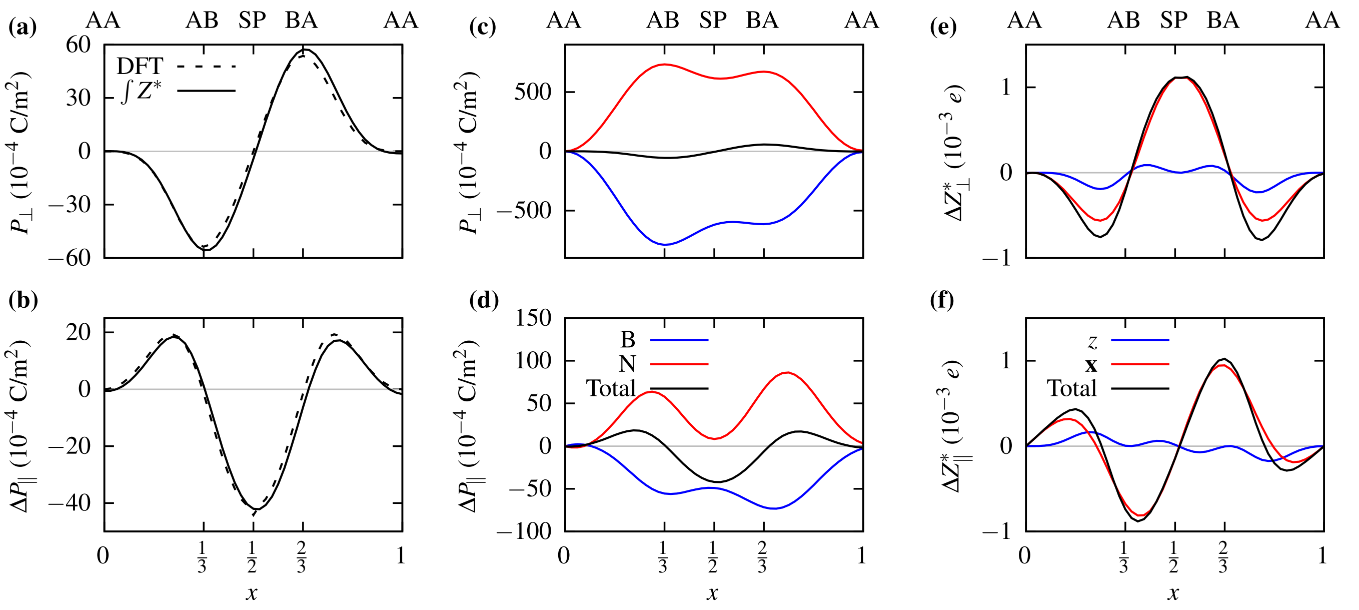

Supplementary FIG. 4. Total polarization in **(a)** the out-of-plane direction and **(b)** the in-plane direction, obtained by integrating the dynamical charges (solid), and from Berry phases (dashed). Individual contributions to the total polarization (black) in **(c)** the out-of-plane direction and **(d)** the in-plane direction, from the displacement of the B (blue) and N (red) atoms. Total change in dynamical charges (black) and the decomposition into out-of-plane (blue) and in-plane (red) displacements in **(e)** the out-of-plane direction and **(f)** the in-plane direction.

The dynamical charges were calculated for bilayer hBN along the diagonal in configuration space using density functional perturbation theory (DFPT) calculations in ABINIT. Integrating the effective charges results in a polarization almost in exact agreement with the polarization obtained by calculating the Berry phases, see Figs. 4 (a) and (b).

One advantage to calculating the polarization from the effective charges is that it allows the decomposition of the total polarization into contributions from the displacement of individual atoms in different directions. In Figs. 4 (c) and (d), the separate contributions to the polarization arising from the displacement of the B and N atoms in the top layer are shown. For the out-of-plane polarization, displacing the two atoms generates large, almost equal but opposite polarizations, which cancel to give the dipole generated by the transferred electronic charge. The individual contributions to the in-plane polarization have the same order of magnitude as the total in-plane polarization. Neither contribution by itself is even, but their sum is even. The total change of the effective charges in the top layer are shown in Figs. 4 (e) and (f), from which it is clear that the sum of the effective charges is the integrand of the total polarization. Notably, the polarization is almost entirely generated from the relative in-plane displacements. Out-of-plane displacements, i.e. the modulation of the interlayer separation, results in a higher-order contribution which is much smaller in magnitude.

V. CONVERGENCE OF THE TOPOLOGICAL CHARGE

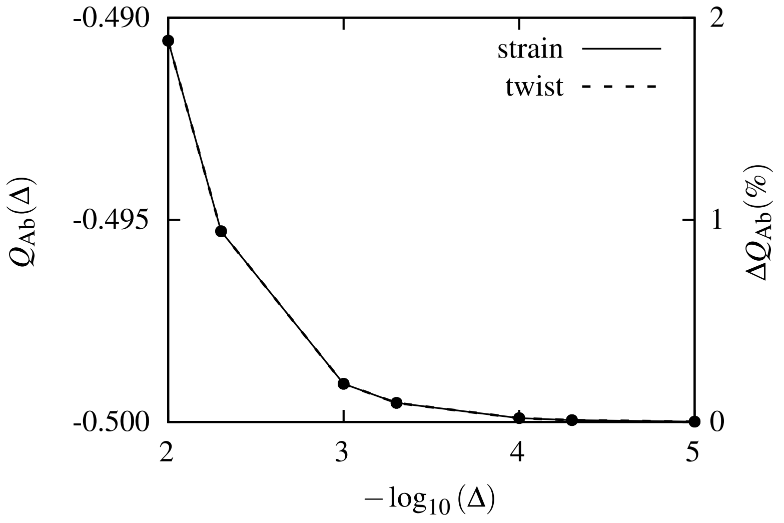

Supplementary FIG. 5. Convergence of the total topological charge / winding number of the AB domain Q_{AB} for a strained (solid line) and twisted (dashed line) bilayer as a function of grid spacing Δ. The second y-axis shows the percentage error.

VI. WINDING ALONG THE CELL BOUNDARIES FOR AN APPLIED ELECTRIC FIELD

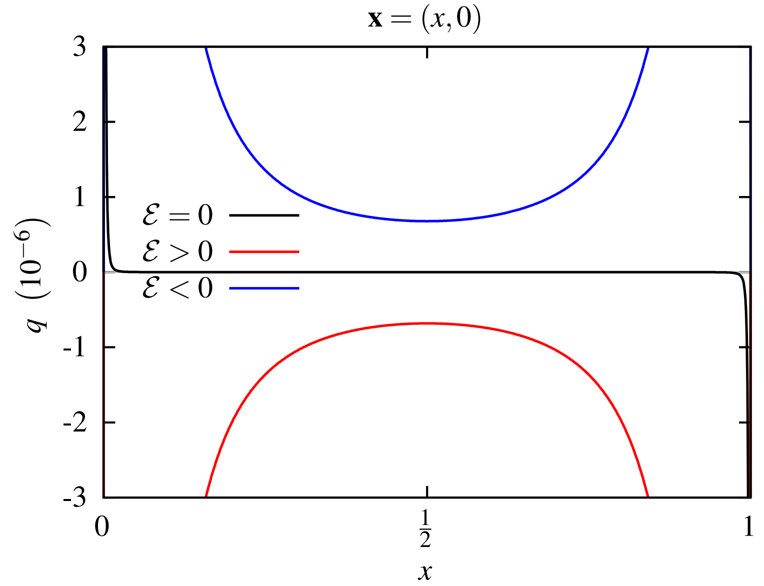

Supplementary FIG. 6. Local winding along $\mathbf{x} = (x, 0)$ in a single moiré cell embedded in a dielectric medium for zero field (black) and for out-of-plane electric fields applied in the positive (red) and negative (blue) directions.

SUPPLEMENTARY REFERENCES

[1] Carr, S. *et al.* Relaxation and domain formation in incommensurate two-dimensional heterostructures. *Phys. Rev. B* **98**, 224102 (2018).

[2] Bennett, D. & Remez, B. On electrically tunable stacking domains and ferroelectricity in moiré superlattices. *npj 2D Mater. Appl.* **6**, 1–6 (2022).

[3] Ghosez, P., Michenaud, J.-P. & Gonze, X. Dynamical atomic charges: The case of ABO_3 compounds. *Phys. Rev. B* **58**, 6224 (1998).
